# *N*-(4-Bromo­phen­yl)-2-(4-chloro­phen­yl)acetamide

**DOI:** 10.1107/S1600536812002383

**Published:** 2012-01-25

**Authors:** Hoong-Kun Fun, Tara Shahani, Prakash S. Nayak, B. Narayana, B. K. Sarojini

**Affiliations:** a X-ray Crystallography Unit, School of Physics, Universiti Sains Malaysia, 11800 USM, Penang, Malaysia; b Department of Studies in Chemistry, Mangalore University, Mangalagangotri 574 199, India; c Department of Chemistry, P.A. College of Engineering, Nadupadavu, Mangalore 574 153, India

## Abstract

The title compound, C_14_H_11_BrClNO, consists of chloro­benzene and bromo­benzene units which are linked at either end of the *N*-methyl­propionamide group. The chloro­benzene unit [maximum deviation = 0.005 (4) Å] makes a dihedral angle of 68.21 (19)° with the bromo­benzene unit [maximum deviation = 0.012 (3) Å]. In the crystal, N—H⋯O hydrogen bonds link the mol­ecules into chains along [010].

## Related literature

For the structural similarity of *N*-substituted 2-aryl­acetamides to the lateral chain of natural benzyl­penicillin, see: Mijin & Marinkovic (2006 ▶); Mijin *et al.* (2008 ▶). For the coordination abilities of amides, see: Wu *et al.* (2008 ▶, 2010 ▶). For related structures, see: Praveen *et al.* (2011*a* ▶,*b* ▶,*c* ▶); Fun *et al.* (2011*a* ▶,*b* ▶).
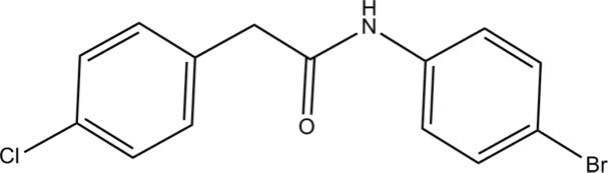


## Experimental

### 

#### Crystal data

C_14_H_11_BrClNO
*M* *_r_* = 324.60
Monoclinic, 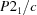

*a* = 15.584 (8) Å
*b* = 4.763 (3) Å
*c* = 18.139 (10) Å
β = 96.984 (11)°
*V* = 1336.5 (12) Å^3^
*Z* = 4
Mo *K*α radiation
μ = 3.26 mm^−1^
*T* = 296 K
0.69 × 0.19 × 0.06 mm

#### Data collection

Bruker SMART APEXII CCD area-detector diffractometer
Absorption correction: multi-scan (*SADABS*; Bruker, 2009 ▶) *T*_min_ = 0.211, *T*_max_ = 0.826
12836 measured reflections
3880 independent reflections
1970 reflections with *I* > 2σ(*I*)
*R*_int_ = 0.076

#### Refinement

*R*[*F* ^2^ > 2σ(*F* ^2^)] = 0.059
*wR*(*F* ^2^) = 0.166
*S* = 1.03
3880 reflections
167 parameters
H atoms treated by a mixture of independent and constrained refinement
Δρ_max_ = 0.52 e Å^−3^
Δρ_min_ = −0.54 e Å^−3^

### 

Data collection: *APEX2* (Bruker, 2009 ▶); cell refinement: *SAINT* (Bruker, 2009 ▶); data reduction: *SAINT*; program(s) used to solve structure: *SHELXTL* (Sheldrick, 2008 ▶); program(s) used to refine structure: *SHELXTL*; molecular graphics: *SHELXTL*; software used to prepare material for publication: *SHELXTL* and *PLATON* (Spek, 2009 ▶).

## Supplementary Material

Crystal structure: contains datablock(s) global, I. DOI: 10.1107/S1600536812002383/tk5050sup1.cif

Structure factors: contains datablock(s) I. DOI: 10.1107/S1600536812002383/tk5050Isup2.hkl

Supplementary material file. DOI: 10.1107/S1600536812002383/tk5050Isup3.cml

Additional supplementary materials:  crystallographic information; 3D view; checkCIF report

## Figures and Tables

**Table 1:** Hydrogen-bond geometry (Å, °)

*D*—H⋯*A*	*D*—H	H⋯*A*	*D*⋯*A*	*D*—H⋯*A*
N1—H1*N*1⋯O1^i^	0.71 (4)	2.17 (4)	2.843 (4)	160 (5)

Symmetry code: (i) 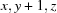.

**Fig. 1.**
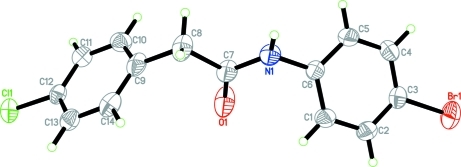
The molecular structure of the title compound, showing 30% probability displacement ellipsoids and the atom numbering scheme.

**Fig. 2.**
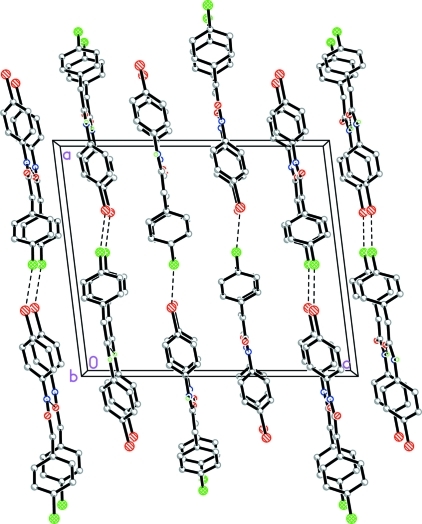
A view of the crystal packing where N1—H1N1···O1 hydrogen bonds (dashed lines) link the molecules into chains along [010]. Hydrogen atoms not involved in hydrogen bonding have been omitted for clarity.

## supplementary crystallographic information

### Comment

*N*-Substituted 2-arylacetamides are very interesting compounds because of their structural similarity to the lateral chain of natural benzylpenicillin (Mijin & Marinkovic, 2006; Mijin *et al.*, 2008). Amides are also used as ligands due to their excellent coordination abilities (Wu *et al.*, 2008, 2010). Crystal structures of some acetamide derivatives viz., *N*-(4-chloro-1,3-benzothiazol-2-yl)-2-(3-methylphenyl) acetamide monohydrate, *N*-(3-chloro-4-fluorophenyl)-2,2-diphenylacetamide and *N*-(3-chloro-4-fluorophenyl)-2-(naphthalen-1-yl)acetamide (Praveen *et al.*, 2011**a*,*b*,c*) have been reported. In continuation of our work on synthesis of amides (Fun *et al.*, 2011**a*,b*) we report herein the crystal structure of the title compound.

The title compound (Fig. 1), consists of a chlorobenzene (C9–C14/Cl1) and bromobenzene (C1–C6/Br1) moieties which are attached to the *N*-methylpropionamide (N1/C7–C8/O1) group. The chlorobenzene moiety (maximum deviations of 0.005 (4) at atom C10) makes dihedral angle of 68.21 (19)° with bromobenzene moiety (maximum deviations of 0.012 (3) Å at atom C6). Bond lengths are comparable to those in related structures (Fun *et al.*, 2011**a*,b*).

In the crystal packing (Fig. 2), intermolecular N1—H1N1···O1 hydrogen bonds link the molecules into chains along [010].

### Experimental

4-Chlorophenylacetic acid (0.170g, 1mmol) and 4-bromoaniline (0.172g, 1mmol), 1-ethyl-3-(3-dimethylaminopropyl)-carbodiimide hydrochloride(1.0g, 0.01mol) and were dissolved in dichloromethane (20mL). The mixture was stirred in presence of triethylamine at 273 K for about 3 h. The contents were poured into 100 ml of ice-cold aqueous hydrochloric acid with stirring, which was extracted thrice with dichloromethane. Organic layer was washed with saturated NaHCO_3_ solution and brine solution, dried and concentrated under reduced pressure to give the title compound (I). Single crystals were grown from dichloromethane mixture by the slow evaporation method *mp*: 439–441 k.

### Refinement

N-bound H atom was located in a difference map and were refind freely. [N–H = 0.71 (4) Å] The remaining H atoms were positioned geometrically and were refined with a riding model with *U*_iso_(H) = 1.2 *U*_eq_(C) [C–H = 0.933, 0.9700 Å].

### Crystal data

**Table:**

C_14_H_11_BrClNO	*F*(000) = 648
*M**_r_* = 324.60	*D*_x_ = 1.613 Mg m^−^^3^
Monoclinic, *P*2_1_/*c*	Mo *K*α radiation, λ = 0.71073 Å
Hall symbol: -P 2ybc	Cell parameters from 1975 reflections
*a* = 15.584 (8) Å	θ = 2.5–24.6°
*b* = 4.763 (3) Å	µ = 3.26 mm^−^^1^
*c* = 18.139 (10) Å	*T* = 296 K
β = 96.984 (11)°	Plate, colourless
*V* = 1336.5 (12) Å^3^	0.69 × 0.19 × 0.06 mm
*Z* = 4	

### Data collection

**Table:**

Bruker SMART APEXII CCD area-detector diffractometer	3880 independent reflections
Radiation source: fine-focus sealed tube	1970 reflections with *I* > 2σ(*I*)
graphite	*R*_int_ = 0.076
φ and ω scans	θ_max_ = 30.0°, θ_min_ = 2.3°
Absorption correction: multi-scan (*SADABS*; Bruker, 2009)	*h* = −21→21
*T*_min_ = 0.211, *T*_max_ = 0.826	*k* = −6→6
12836 measured reflections	*l* = −25→24

### Refinement

**Table:**

Refinement on *F*^2^	Primary atom site location: structure-invariant direct methods
Least-squares matrix: full	Secondary atom site location: difference Fourier map
*R*[*F*^2^ > 2σ(*F*^2^)] = 0.059	Hydrogen site location: inferred from neighbouring sites
*wR*(*F*^2^) = 0.166	H atoms treated by a mixture of independent and constrained refinement
*S* = 1.03	*w* = 1/[σ^2^(*F*_o_^2^) + (0.*P*)^2^ + 0.5271*P*] where *P* = (*F*_o_^2^ + 2*F*_c_^2^)/3
3880 reflections	(Δ/σ)_max_ < 0.001
167 parameters	Δρ_max_ = 0.52 e Å^−^^3^
0 restraints	Δρ_min_ = −0.54 e Å^−^^3^

### Special details

**Table:**

Geometry. All esds (except the esd in the dihedral angle between two l.s. planes) are estimated using the full covariance matrix. The cell esds are taken into account individually in the estimation of esds in distances, angles and torsion angles; correlations between esds in cell parameters are only used when they are defined by crystal symmetry. An approximate (isotropic) treatment of cell esds is used for estimating esds involving l.s. planes.
Refinement. Refinement of F^2^ against ALL reflections. The weighted R-factor wR and goodness of fit S are based on F^2^, conventional R-factors R are based on F, with F set to zero for negative F^2^. The threshold expression of F^2^ > 2sigma(F^2^) is used only for calculating R-factors(gt) etc. and is not relevant to the choice of reflections for refinement. R-factors based on F^2^ are statistically about twice as large as those based on F, and R- factors based on ALL data will be even larger.

### Fractional atomic coordinates and isotropic or equivalent isotropic displacement parameters (Å^2^)

**Table:**

	*x*	*y*	*z*	*U*_iso_*/*U*_eq_	
Br1	0.28762 (3)	0.70007 (12)	0.35364 (3)	0.0891 (3)	
O1	−0.1362 (2)	0.6167 (5)	0.3893 (3)	0.1018 (14)	
N1	−0.0827 (2)	1.0487 (6)	0.37965 (19)	0.0562 (9)	
Cl1	−0.52585 (7)	0.1650 (2)	0.38444 (7)	0.0663 (3)	
C1	0.0462 (3)	0.7777 (7)	0.4237 (2)	0.0594 (10)	
H1A	0.0171	0.6980	0.4604	0.071*	
C2	0.1300 (3)	0.7001 (8)	0.4179 (2)	0.0607 (10)	
H2A	0.1574	0.5677	0.4504	0.073*	
C3	0.1732 (3)	0.8182 (8)	0.3642 (2)	0.0573 (9)	
C4	0.1331 (3)	1.0177 (8)	0.3172 (2)	0.0667 (11)	
H4A	0.1627	1.1011	0.2814	0.080*	
C5	0.0495 (3)	1.0933 (8)	0.3232 (3)	0.0652 (11)	
H5A	0.0226	1.2285	0.2914	0.078*	
C6	0.0047 (2)	0.9718 (6)	0.3758 (2)	0.0471 (8)	
C7	−0.1479 (3)	0.8708 (6)	0.3853 (2)	0.0597 (10)	
C8	−0.2359 (3)	1.0010 (7)	0.3854 (3)	0.0756 (14)	
H8A	−0.2475	1.1210	0.3421	0.091*	
H8B	−0.2354	1.1186	0.4291	0.091*	
C9	−0.3081 (3)	0.7905 (7)	0.3849 (3)	0.0626 (12)	
C10	−0.3510 (3)	0.6898 (7)	0.3188 (3)	0.0634 (11)	
H10A	−0.3350	0.7532	0.2740	0.076*	
C11	−0.4175 (3)	0.4957 (7)	0.3185 (2)	0.0578 (9)	
H11A	−0.4465	0.4305	0.2739	0.069*	
C12	−0.4396 (2)	0.4024 (7)	0.3850 (2)	0.0491 (9)	
C13	−0.3986 (3)	0.4935 (8)	0.4515 (2)	0.0577 (9)	
H13A	−0.4145	0.4264	0.4960	0.069*	
C14	−0.3332 (3)	0.6879 (8)	0.4508 (3)	0.0625 (11)	
H14A	−0.3049	0.7522	0.4957	0.075*	
H1N1	−0.087 (3)	1.197 (8)	0.376 (2)	0.058 (12)*	

### Atomic displacement parameters (Å^2^)

**Table:**

	*U*^11^	*U*^22^	*U*^33^	*U*^12^	*U*^13^	*U*^23^
Br1	0.0498 (3)	0.1141 (5)	0.1047 (5)	0.0028 (2)	0.0147 (3)	−0.0110 (3)
O1	0.0549 (17)	0.0276 (11)	0.223 (4)	0.0063 (11)	0.018 (2)	−0.0026 (17)
N1	0.055 (2)	0.0268 (13)	0.086 (2)	0.0063 (12)	0.0059 (17)	−0.0021 (13)
Cl1	0.0496 (6)	0.0703 (6)	0.0804 (8)	−0.0048 (4)	0.0137 (5)	−0.0103 (5)
C1	0.059 (2)	0.0541 (19)	0.067 (3)	0.0103 (16)	0.011 (2)	0.0114 (17)
C2	0.057 (2)	0.060 (2)	0.065 (3)	0.0104 (17)	0.004 (2)	0.0087 (18)
C3	0.048 (2)	0.058 (2)	0.065 (3)	−0.0049 (16)	0.005 (2)	−0.0097 (18)
C4	0.062 (3)	0.073 (2)	0.066 (3)	−0.014 (2)	0.013 (2)	0.012 (2)
C5	0.064 (3)	0.0519 (19)	0.076 (3)	−0.0053 (18)	−0.004 (2)	0.0184 (18)
C6	0.0466 (19)	0.0307 (14)	0.063 (2)	0.0002 (13)	0.0010 (17)	−0.0045 (14)
C7	0.055 (2)	0.0294 (14)	0.094 (3)	0.0085 (14)	0.006 (2)	−0.0064 (16)
C8	0.052 (2)	0.0350 (16)	0.140 (4)	0.0064 (15)	0.011 (3)	−0.009 (2)
C9	0.047 (2)	0.0377 (16)	0.102 (4)	0.0123 (14)	0.006 (2)	−0.0054 (18)
C10	0.070 (3)	0.0524 (19)	0.069 (3)	0.0048 (18)	0.010 (2)	0.0058 (18)
C11	0.058 (2)	0.057 (2)	0.058 (2)	0.0030 (17)	0.0024 (19)	−0.0067 (17)
C12	0.0416 (19)	0.0458 (17)	0.060 (2)	0.0098 (14)	0.0067 (18)	−0.0048 (15)
C13	0.050 (2)	0.068 (2)	0.054 (2)	0.0101 (17)	0.0044 (18)	−0.0076 (18)
C14	0.055 (2)	0.060 (2)	0.070 (3)	0.0101 (18)	−0.003 (2)	−0.0201 (19)

### Geometric parameters (Å, °)

**Table:**

Br1—C3	1.902 (4)	C5—H5A	0.9300
O1—C7	1.225 (4)	C7—C8	1.506 (5)
N1—C7	1.337 (5)	C8—C9	1.506 (5)
N1—C6	1.421 (5)	C8—H8A	0.9700
N1—H1N1	0.71 (4)	C8—H8B	0.9700
Cl1—C12	1.756 (4)	C9—C10	1.384 (6)
C1—C2	1.374 (6)	C9—C14	1.391 (6)
C1—C6	1.374 (5)	C10—C11	1.388 (6)
C1—H1A	0.9300	C10—H10A	0.9300
C2—C3	1.370 (6)	C11—C12	1.369 (5)
C2—H2A	0.9300	C11—H11A	0.9300
C3—C4	1.376 (6)	C12—C13	1.364 (5)
C4—C5	1.369 (6)	C13—C14	1.379 (6)
C4—H4A	0.9300	C13—H13A	0.9300
C5—C6	1.376 (5)	C14—H14A	0.9300
			
C7—N1—C6	125.6 (3)	C7—C8—C9	113.9 (3)
C7—N1—H1N1	125 (4)	C7—C8—H8A	108.8
C6—N1—H1N1	109 (4)	C9—C8—H8A	108.8
C2—C1—C6	120.7 (4)	C7—C8—H8B	108.8
C2—C1—H1A	119.6	C9—C8—H8B	108.8
C6—C1—H1A	119.6	H8A—C8—H8B	107.7
C3—C2—C1	119.8 (4)	C10—C9—C14	117.7 (4)
C3—C2—H2A	120.1	C10—C9—C8	121.2 (4)
C1—C2—H2A	120.1	C14—C9—C8	121.1 (4)
C2—C3—C4	120.0 (4)	C9—C10—C11	121.1 (4)
C2—C3—Br1	119.9 (3)	C9—C10—H10A	119.4
C4—C3—Br1	120.2 (3)	C11—C10—H10A	119.4
C5—C4—C3	119.8 (4)	C12—C11—C10	118.6 (4)
C5—C4—H4A	120.1	C12—C11—H11A	120.7
C3—C4—H4A	120.1	C10—C11—H11A	120.7
C4—C5—C6	120.8 (4)	C13—C12—C11	122.4 (4)
C4—C5—H5A	119.6	C13—C12—Cl1	119.1 (3)
C6—C5—H5A	119.6	C11—C12—Cl1	118.6 (3)
C1—C6—C5	118.8 (4)	C12—C13—C14	118.2 (4)
C1—C6—N1	121.5 (3)	C12—C13—H13A	120.9
C5—C6—N1	119.7 (3)	C14—C13—H13A	120.9
O1—C7—N1	121.5 (3)	C13—C14—C9	122.0 (4)
O1—C7—C8	122.4 (3)	C13—C14—H14A	119.0
N1—C7—C8	116.1 (3)	C9—C14—H14A	119.0
			
C6—C1—C2—C3	0.4 (6)	O1—C7—C8—C9	−4.4 (7)
C1—C2—C3—C4	1.2 (6)	N1—C7—C8—C9	174.7 (4)
C1—C2—C3—Br1	−177.5 (3)	C7—C8—C9—C10	−89.3 (5)
C2—C3—C4—C5	−1.4 (6)	C7—C8—C9—C14	89.9 (5)
Br1—C3—C4—C5	177.3 (3)	C14—C9—C10—C11	0.8 (5)
C3—C4—C5—C6	−0.1 (6)	C8—C9—C10—C11	180.0 (3)
C2—C1—C6—C5	−1.8 (6)	C9—C10—C11—C12	−0.7 (5)
C2—C1—C6—N1	178.2 (3)	C10—C11—C12—C13	0.1 (5)
C4—C5—C6—C1	1.7 (6)	C10—C11—C12—Cl1	178.1 (3)
C4—C5—C6—N1	−178.4 (4)	C11—C12—C13—C14	0.4 (5)
C7—N1—C6—C1	−46.6 (6)	Cl1—C12—C13—C14	−177.6 (3)
C7—N1—C6—C5	133.5 (4)	C12—C13—C14—C9	−0.3 (5)
C6—N1—C7—O1	1.5 (7)	C10—C9—C14—C13	−0.3 (5)
C6—N1—C7—C8	−177.6 (4)	C8—C9—C14—C13	−179.5 (3)

### Hydrogen-bond geometry (Å, °)

**Table:**

*D*—H···*A*	*D*—H	H···*A*	*D*···*A*	*D*—H···*A*
N1—H1N1···O1^i^	0.71 (4)	2.17 (4)	2.843 (4)	160 (5)

Symmetry codes: (i) *x*, *y*+1, *z*.
